# On-field rehabilitation in football: Current knowledge, applications and future directions

**DOI:** 10.3389/fspor.2022.970152

**Published:** 2022-12-05

**Authors:** Mark Armitage, Stuart A. McErlain-Naylor, Gavin Devereux, Marco Beato, Matthew Buckthorpe

**Affiliations:** ^1^School of Health and Sports Sciences, University of Suffolk, Ipswich, United Kingdom; ^2^Performance Services Department, Norwich City Football Club, Norwich, United Kingdom; ^3^Faculty of Sport, Allied Health and Performance Science, St Mary's University Twickenham, London, United Kingdom; ^4^School of Sport, Exercise and Health Sciences, Loughborough University, Loughborough, United Kingdom

**Keywords:** field-based, rehab, soccer, injury, re-conditioning, prevention

## Introduction

Injury reduction remains a hot topic in professional football due to the economic and competitive implications of time lost (1, 2). Current strategies to reduce injury burden involve either reducing primary injuries through prevention-based strategies or lowering the risk of secondary injuries when they occur. It appears that primary injury reduction strategies are largely effective (3, 4), and might have supported reduced incidence across the past two decades (5, 6). Strategies concerning re-injury risk, however, are less than optimal, particularly when concerning recurrent and/or high-grade muscle and ligament injuries (1, 5). Whilst return to play (RTP) rates for such injuries are high in elite football, players often return with heightened risk of re-injury and may experience lower performance levels, especially after severe injuries such as anterior cruciate ligament (ACL) ruptures (7–14). Injuries are thought to occur due to a complex web of determinants (15), with previous injury remaining one of the most reported risk factors (16). Re-injuries (i.e., to the same location) or subsequent injuries (i.e., in a different location) typically occur early in the RTP process, suggesting players might be returned too quickly for sufficient tissue healing, or they are inadequately prepared for RTP demands (6, 16–18). The role of previous injury as a risk factor for future injury can be mitigated through effective rehabilitation (19). As such, improving RTP practice and processes appears warranted to improve outcomes after certain injuries (e.g., high-grade muscle/severe ligament injuries).

**Figure 1:**
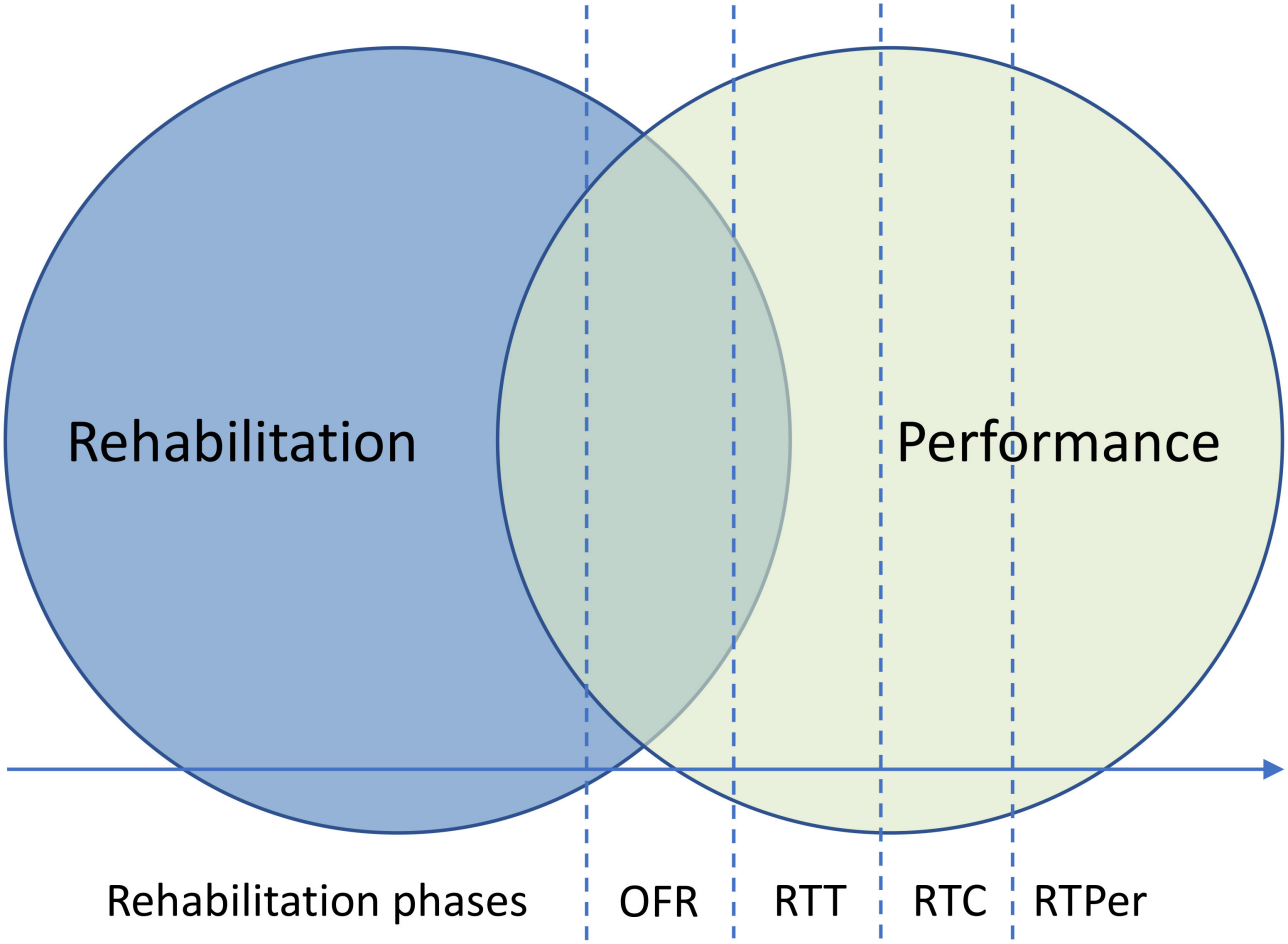
A return to sport process involving a gradual transition from rehabilitation to performance training along a continuum of OFR, RTT, RTC, and RTPer. ORF, on-field rehabilitation; RTT, return to training; RTC, return to competition; RTPer, return to performance. Modified and re-printed with permission from Buckthorpe et al. (21).

There is a lack of consensus on effective rehabilitation for such injuries, with current evidence suggesting that players should embark on a criterion-based process through a series of stages (20). These typically include early-, mid- and late-stage rehabilitation, followed by a RTP continuum, involving on-field rehabilitation (OFR), return to team training, return to competitive match-play and finally a return to performance (Figure 1) (21–26). Recently, there has been an increase in translational research published to support football medicine departments with their late-stage rehabilitation processes, specifically that of OFR (21, 22, 26, 27). OFR as a service is not new with numerous practitioners establishing unpublished frameworks before evidence-based practice and load monitoring technologies existed. Scientific developments however have facilitated two separate published frameworks for OFR, which use competency-based continua to provide evidential structures to support long-established practices (21, 22, 26). Despite improving clarity, such research is currently restricted to expert opinion and/or case studies. Although this is a complex topic with numerous inherent challenges, future research should attempt validation of such frameworks.

The purpose of this article is to (i) review injury incidence literature to assess the prevalence of re-injuries and postulate OFR as a potential tool to mitigate future risk, (ii) consider injury aetiology and the complexity of OFR, (iii) describe existing OFR frameworks, and (iv) offer future directions related to the development of OFR in professional football.

## Injury outcomes, (re-) injury epidemiology, and the importance of on-field rehabilitation

Understanding injury occurrence, healing timeframes and RTP rates are vital when designing, implementing, and evaluating OFR frameworks. When injuries occur, they are often categorised based on their severity, or the potential for time loss. Most injuries are mild (≤7 days), and overall RTP rates from all injuries are high, however those returning from severe injuries (>28 days) such as ACL ruptures often face long absence, elevated re-injury risk and reduced performance levels (1, 9). Overall, injuries have reduced by ~3% per year over the past 18 years, with muscle injury rates remaining unchanged (5). Although this should be considered in the context of greater frequencies and intensities of matches nowadays, muscle injuries remain a concern given their susceptibility to re-injury (17, 28). Indeed, injuries involving musculature of the lower limbs remain notable (~15%) (1).

Ekstrand et al. (1) reported ACL re-injury rates at 6.6%, which is in-keeping with others (29), but less than the 18% reported by Della Villa et al. (9). However, it is perhaps severity and not incidence which is of concern for ACL injuries, with a mean absence of 205 days (1). Although, re-injury rates were low in the study of Waldén et al. (29), five out of the nine re-ruptures occurred during the final phase of rehabilitation or before the first match, and all others were within the first 3 months after the first match. The timing of these re-injuries suggests an increased risk during on-field activities and reinforces the importance of effective OFR frameworks.

## Injury aetiology and the complex nature of on-field rehabilitation

All injuries are related to an overload of some type, whether they involve trauma (i.e., contact), mechanical failure (i.e., non-contact) or a combination of both (i.e., indirect contact) (30, 31). They occur when the stress and/or strain on the body tissue exceeds the maximal strength or failure strain of that tissue (32). Injury prevention models have traditionally been based on a reductionist view (15, 33) that simplifies multifaceted components into units, attempting to identify relationships and sequence events (e.g., isolating the mechanism, site, type, and treatment of injury) (34, 35). In reality, injury involves complex interactions between numerous factors, and so seemingly comparable situations may yield different outcomes (15). Contributing factors might include any combination of neural inhibition, selective muscle atrophy, alterations in fascicle length, strength deficits and/or increased susceptibility to fatigue, amongst others (36). A holistic approach to rehabilitation is therefore required to accommodate the complex and individual nature of the process. OFR is considered a vital component, due to the ecological validity offered by manipulating various training stimuli to stimulate tissue loading in a manner which more closely resembles that experienced during training and competition (37).

Football matches are now played at a greater frequency and intensity than ever before, which increases the physiological and mechanical demands on players (5, 38). This emphasises the need for players to be appropriately re-conditioned to RTP (18). Despite research warning that an imbalance in “load” between rehabilitation and match-play might increase the risk of re-injury (17), specific information is sparse (18). Whilst any relationship between “training load” and injury is likely to be associative and not definitively causative (39), clear aetiology is yet to be established (40). Researchers and practitioners are interested in exercise volume and intensity, and the external and internal “loads” associated to these (41, 42). To improve understanding, there is need for agreement over terms and technology used to describe and measure discrete outputs. For now, multiple independent metrics are required during OFR (e.g., running distance and velocity; step frequency, intensity, and symmetry; heart rate; and rating of perceived exertion), considering both the psycho-physiological and mechanical aspects of load-adaptation pathways (38, 40, 43).

## Existing return to play frameworks and the developing role of on-field rehabilitation

To aid decision-making during rehabilitation, Creighton et al. (44) developed a three-step model: Step 1—evaluation of health status in consideration with medical factors; Step 2—evaluation of participation risk in consideration with sport risk modifiers; and Step 3—decision modification in consideration with decision modifiers. Step 1 is arguably the most clinically important because it indicates the state of healing and thus enables risk-assessment decision-making. These decisions are also task-specific (Step 2). For example, the risk associated with an upper limb injury for an outfield player will differ to that posed by the same injury to a goalkeeper. Finally, non-medical factors (Step 3), such as time in season, external influences, and conflicts of interest, need to be considered to provide context to decision-making (44). Whilst this model provided a framework to inform decisions based on the assessment of multiple risk factors, concerns were raised with regards to limitations and implementation (45).

The model was modified accordingly to form the Strategic Assessment of Risk and Risk Tolerance (StARRT) framework (45). The structure remained the same, but the terminology was updated alongside the ordering of contributing factors. Although the StARRT framework was included in the 2016 consensus statement on RTP (46), the statement suggested combining biopsychosocial factors with continued application and evaluation of the framework. Where possible, shared decision-making between the player, practitioner and appropriate others should also take place (47). Practitioners should use the available evidence and their own experiences, combined with knowledge of the individual, specific scenario, and club philosophy, to shape their RTP protocols (48). An evidence-based approach to decision-making has recently been enhanced for football through the development of two specific OFR frameworks (21, 22, 26).

Buckthorpe et al. (21) offer a four-pillar structure for practitioners to plan their on-field progressions: 1—movement quality; 2—physical conditioning; 3—sport-specific skills; 4—training load. Restoration of movement patterns should be addressed first, before increasing metabolic and mechanical demands and then integrating neurocognitive and perceptual challenges to enhance specificity. Once the player has increased confidence in the injury site, often in one-to-one environments, they can begin re-introduction to team-based interactions and the club's conditioning model. The four pillars have been additionally described as contributing to a five stage OFR process (after ACL injury): 1—linear movement; 2—multidirectional movement; 3—soccer-specific technical skills; 4—soccer-specific movements; and 5—practice simulation (22). Whilst this framework was designed as an educational piece to support practitioners in structuring their OFR processes, currently there is little evidence of usage or effectiveness.

Taberner et al. (26) offer a similar five stage framework, eloquently titled the control-chaos continuum: 1—high control; 2—moderate control; 3—control to chaos; 4—moderate chaos; 5—high chaos. Progressing sport-specific physical conditioning, technical skills and movement qualities, practitioners are encouraged to systematically manipulate volume and intensity whilst increasing uncertainty of action. This framework has been applied through a series of elite player case studies including a male tibia-fibula fracture (49), female ACL reconstruction (50), and male semimembranosus reconstruction (51). Whilst the stages remained the same for each case, durations were altered to reflect the specific needs of each injury.

Both frameworks position OFR as competency-based and not just time dependent (21, 22, 26). However, there remains a lack of validated competency criteria for RTP protocols (1). Whilst both frameworks act as a reference guide for practitioners and facilitate future research processes, they are based on existing theory, experience, and inductive reasoning (52). Experimental studies utilising hypothesis testing to promote validation are now needed (53).

Jimenez-Rubio and colleagues attempt to provide some evidence by using an expert panel to gain agreement for an on-field readaptation programme following a hamstring injury (54), and a rehabilitation and reconditioning programme following an adductor longus injury (55). These authors performed a follow-up study with those who completed the hamstring protocol and reported that not only had the injury site fully recovered, but following rehabilitation players could withstand greater match and training demands, with a reduced risk of future injury (56). Whilst this highlights the importance of OFR and improving evidential structures, the 13-item OFR programme (54) is quite prescriptive and could be challenged given the individual nature of injuries and responses to interventions. Conceptual frameworks such as the control-chaos continuum and four-pillars of on-field rehabilitation may offer greater flexibility. In essence, frameworks should support and not dictate decision-making, with practitioners and researchers empowered to continually evolve their practice and understanding.

Regardless of which conceptual framework is used, it is recommended that players progress systematically to develop load tolerance of the injury site and restore sport specific qualities (21, 22, 26). Whilst the control-chaos continuum places a greater emphasis on cognitive demands as progressions become more “chaotic”, both frameworks promote “load” progression/management. Improved understanding of the “load” requirements of specific drills/activities within each stage and potential progression targets between stages, would support the development of either framework.

## Areas for future research

Although the frameworks use different terminology, they both offer stepwise OFR progressions to practitioners. Agreement in terminology would be useful to enhance application, as would research into specific “load” responses to explore which drills typically fall into which stages. Currently, there is no substantial advice on how to specifically measure and progress OFR (57). Whilst progressions within and between sessions and stages in the available frameworks appear rational, they are yet to be empirically established. Training “load” appears to be a key determinant in effective OFR (18, 21, 22, 26, 58), therefore the development of specific sessional content (i.e., drill level analysis) should further support practitioners in their decision-making (59). As OFR is not a new concept, current practice with regards to drill/activity selection (including input from technical coaches who should be active drill designers) should be explored to identify potential gaps and enhance application of future findings (27). These drills/activities can then be investigated using a range of monitoring techniques (e.g., heart rate, global position systems, inertial measurement units, and rating of perceived exertion, amongst others) to measure some of the psycho-physiological and mechanical demands. Currently, knowledge of causality between training “load” application and successful RTP outcomes is lacking. Future research can use the conceptual frameworks mentioned within this article to generate testable hypotheses relating to the outcomes of specific OFR drills/activities associated with the specified stages.

## Summary and implications for practice

Injuries in football, particularly involving muscles and ligaments of the lower limbs, remain problematic, with the risk of secondary (re- or subsequent) injury remaining high. Whilst these often occur within the first few months, risk can remain elevated for years to come. Although epidemiological data are supporting practitioners in targeting injury reduction strategies, previous injury remains one of the largest risk factors for future injury. This highlights the importance of effective rehabilitation protocols when injuries occur, with OFR promoted as a vital bridge between clinical rehabilitation and return to performance. Two conceptual frameworks offer progressive stages for OFR. Whilst these frameworks appear conceptually sound, empirical evidence in this area is lacking. Researchers should work together to find agreement and improve scientific understanding. Drill level analysis, using a range of monitoring techniques to reflect psycho-physiological and mechanical demands, would offer greater insights into within and between session progressions, in turn improving understanding and application of current OFR protocols. Findings should be critically appraised and applied by practitioners to facilitate continued development of evidence-based practice.

## Author contributions

MA was responsible for the concept and writing of this paper. SM-N, GD, MBe, and MBu provided supervision and feedback throughout. All authors contributed to the article and approved the submitted version.

## Conflict of interest

The authors declare that the research was conducted in the absence of any commercial or financial relationships that could be construed as a potential conflict of interest.

## Publisher's note

All claims expressed in this article are solely those of the authors and do not necessarily represent those of their affiliated organizations, or those of the publisher, the editors and the reviewers. Any product that may be evaluated in this article, or claim that may be made by its manufacturer, is not guaranteed or endorsed by the publisher.
